# Address

**Published:** 1877-05

**Authors:** A. T. Metcalf


### THE DENTAL REGISTER.

VOL. XXXI.] **MAY, 1877.** No. 5.

### ADDRESS

*To the Graduating Class, University of Michigan, March* 28, 1877.

BY DR. A. T. METCALF.

YOUNG GENTLEMEN: The degree of Doctor of Dental Surgery has now been conferred on each of you. Permit me to remind you that the diplomas you hold are something more than mere parchment. They have a fourfold significance which is full of interest. They bear witness to time well spent and to labor well directed on your part. They are exponents of the patient, wise and skillful teaching of your professors. They proclaim the intelligent and conservative care of the State, for the welfare of its people. They are your passports to the ranks and the recognition of the dental profession. Fairly won by your own efforts, you have a right to be proud of them. Your teachers sympathize in your pride; the State approves it; and the profession welcome it as evidence of a laudable ambition in your chosen career.

On me has been devolved the grateful duty of welcoming you, on behalf of the dental profession, to its ranks, and to join with your teachers and personal friends, in bidding you God speed in the practical application of the knowledge and skill you have acquired.

Professions and occupations, like the individual men that fill them, are valued according to their usefulness and the benefits they confer on the human race. The dental profession began to be known and recognized as a profession only some thirty-five years ago; but the zeal, the ability, the ingenuity and the practical wisdom of its members have been so conspicuously demonstrated, and the benefits bestowed upon suffering humanity have been so clearly manifested, that, within the lifetime of a single generation the profession has achieved an enviable reputation and an honored position. But do not forget that this proud position of the profession is due to its individual members. Forty years ago the so-called "tooth puller" was a by word. Earnest students, scientific investigators, skillful artists and hard workers have first made *themselves* honorable, and by their associated efforts have made the profession honorable. Such is now its standing in the public estimation, that admission to its recognized ranks *confers honors* on its new members. To a certain extent, you, upon taking your degree, receive an honor which has been won by others, and it now remains for you to consider whether you will be content with this *reflected* honor, or whether by diligent investigation and earnest work you will win *laurels of your own*, and add honor to a profession that first honored you. Do not misunderstand me. In extending to you a hand of professional fellowship, and of cordial welcome to our ranks, you are welcomed to a field whose successful cultivation requires close study, careful investigation, conscientious fidelity, honorable competition and hard work. For it has been wisely ordered, not by our profession, but by the providence that rules and governs all things, that a man, "by the sweat of his brow," shall win, not only the bread that he eats, but the honor that he justly wears.

Believing that you fully comprehend and realize these essential conditions of an honorable success, I join with your scholastic teachers, with these official representatives of the State, and with this "great cloud of witnesses," in wishing you abundant success and prosperity in your chosen career.

I have already spoken of the varied significance of your diploma. Allow me again to allude to it for the purpose of calling your attention, in this connection, to the fact, that the degree this day conferred on each of you, marks a phase or period in your professional life whose precise significance and relation to your future life, it is important you should fully understand and clearly appreciate. To what extent do your diploma and the degree conferred by it, make you dentists? Drawing from your professional knowledge a figure which will convey my thought, I answer: By your *matriculation*, the dental department of this University became your professional alma mater. If that matriculation may be said to have been your professional *birth*, and if the instruction and experience of your scholastic course may be called the years of your professional *childhood*, then, with equal propriety, the dipolma, when conferred on the professional student of this, or any other department, may be said to mark the completion of his *first* professional *dentition*. To complete the figure and my thought—let me add that, by his own incisive first efforts in the practice of his profession, he will be expected to develop his permanent incisors, and in due time his professional molars, to be succeeded by a consciousness (which will be a suprise to himself) that he is just beginning to "cut" his professional "eye teeth;" and when years have passed in that practice and experience which alone can make the knowledge here acquired, *thoroughly his own*, will he be expected to have developed the *dentes sapientiae* which complete the full professsonal dentition. None of us should forget or be unmindful of the fact, my friends, "that life is short and art is long;" and that a man in the climacteric of his years and of his physical development may be and generally is, but a youth in knowledge. The humility, born of the consciousness of this fact, is an essential prerequisite to the ardent and successful pursuit after knowledge and wisdom, and, for this reason, it is also the necessary percursor of the highest honor and the truest success.

Even if time and the proprieties of this occasion permitted, it would be presumptous in me to attempt, in the presence of your distinguished dental professors, to discourse to you on any of the practical duties of the profession you are about to enter. I will therefore content myself with presenting for your consideration one field and department of dentistry in which we are all —even the best of us—students and workers. I do this because I wish, while welcoming you as co-laborers with us in dental pursuits to stimulate you to studies and labors whose possible results may not only crown your own names with honor, but which will also aid us, your professional brethren, and add greatly to the benefits which the profession of dentistry confers on mankind.

As the physician who *cures* a disease is worthy of less honor than he who prevents it—and as the surgeon who *amputates* a limb however skillfully, is indefinitely below him who *saves* it —so my friends, the dentist, who skillfully pulls or plugs a tooth, has lower rank in the profession than he who by preventing decay saves it from either operation.

As in the great department of medicine its highest and ablest men have come to feel the force of the old maxim that "prevention is better than cure," and to make what is called preventive medicine a department, and the very highest department of the science, so in ours; the dentist whose science and skill will teach us to prevent the diseases which give occasion for our art, will be entitled to our highest honor, and the world's richest rewards.

To learn how to correct abnormal and destructive tendencies, to combat the inroads of disease and decay, and to restore to health and usefulness those organs of the mouth which are poisoning the body, torturing the nerves, and disgracing the oral cavity, will furnish employment for your leisure moments, and earn in time, the gratitude of your patrons. To this end, you should devote your best care and skill to children during the eruption and development of their permanent teeth. Even though the child, when grown, may forget your service, the fond parent will recognize and appreciate it. And whether we look at this department of our labors with an eye to pecuniary reward, or to that higher reward which the conscious exercise of skill always brings, I know of no part of dental practice which promises happier results to your patients or more complete satisfaction to yourself.

Nor would I have you halt here. The entire profession have before them the solving of a yet more complicated and important problem. It is the province as well as the privilege of the dentist, in conjunction with the medical physiologist and pathologist, to go yet deeper into the mysteries of cause and cure, until we are able, by the systemic treatment, or the hygienic management of both parent and child, to correct disordered nutrition; and to the end, that the teeth of the child, as well as the rest of its bony system, may be supplied with whatever they lack; or, that the liabilty of the teeth to destruction and decay may be reduced to its lowest possible minimum. Although this idea may, to some, seem, utopian, or like an effort to establish a dental millenium (in which dentists will not be needed), I nevertheless set it before you, young gentlemen, as a field in which you may profitably as well as honorably labor. It is as much our duty, as it is that of the physician, to seek to prevent the very diseases which it is our art to cure. And however perfect and expert you may become in the art of mechanical dentistry, you will find higher honors and richer rewards in striving to so cultivate and advance the science of your profession that, if you live to see the time when "swords shall be beaten into plow-shares and spears into pruning hooks," you will also be ready to convert forceps into fire-tongs and drills into darning needles.

